# Morphological and Compositional Evolution of Oxidative Coke Deposits Layers Generated by Aviation Kerosene

**DOI:** 10.3390/molecules31071218

**Published:** 2026-04-07

**Authors:** Xinyan Pei, Sihan Zou, Keyan Zhang, Zengqi Zhou, Lingyun Hou

**Affiliations:** Institute for Aero Engine, Tsinghua University, Beijing 100084, China; zsh23@mails.tsinghua.edu.cn (S.Z.); zhangkey22@mails.tsinghua.edu.cn (K.Z.); zhou-zq21@mails.tsinghua.edu.cn (Z.Z.); lyhou@tsinghua.edu.cn (L.H.)

**Keywords:** aviation kerosene, oxidative coking, dissolved oxygen, deposit morphology, fuel thermal management

## Abstract

Thermal–oxidative coking of aviation fuel remains a critical limitation for fuel-cooled aero-engine systems operating under high heat loads. This study systematically investigates the oxidative coking behavior of RP-3 aviation kerosene, focusing on the coupled evolution of deposit morphology, composition, and operating conditions. Experiments were conducted in an electrically heated stainless-steel tube while independently varying dissolved oxygen concentration, fuel temperature, temperature gradient, operating pressure, and heating duration. Deposit layers were characterized by SEM and XPS, and residual fuel chemistry was analyzed using GC/MS. The results show that dissolved oxygen governs both the extent and mechanism of coking in the autoxidation regime (150–450 °C). Normal and elevated oxygen levels promote autoxidation of straight-chain alkanes, generating oxygen-containing intermediates that form flocculent, oxygen-rich deposits, whereas near-deoxygenated conditions suppress autoxidation but sustain sulfur-dominated, needle-like deposits. Temperature primarily controls deposition rate and morphology, with steep temperature gradients inducing localized coke formation, while pressure exerts only a minor indirect influence. Prolonged operation leads to deposit densification and non-linear accumulation behavior. These findings clarify the links between fuel chemistry, thermal conditions, and deposit architecture, providing a basis for morphology-aware coking models in fuel-cooled aero-engine systems.

## 1. Introduction

**Figure 1:**
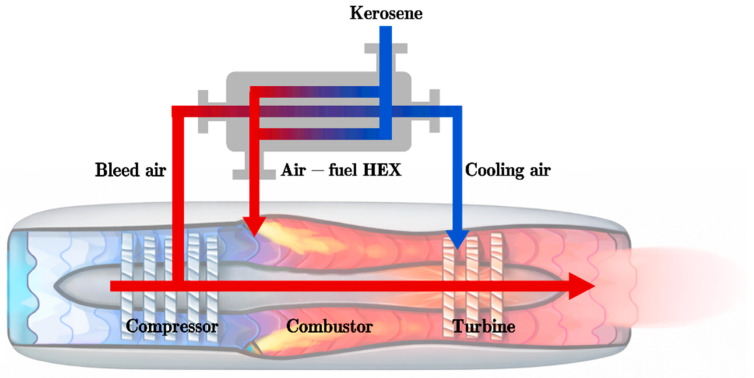
Cooled Cooling Air (CCA) Working Process.

**Figure 2:**
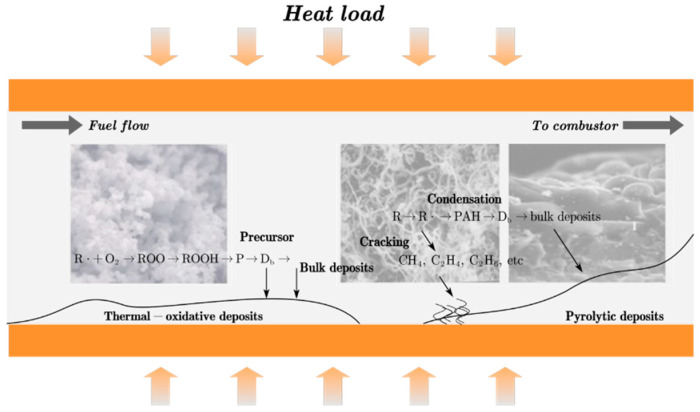
A diagram summarizing oxidative coke deposition.

To maximize the thermal efficiency of modern aircraft gas turbines, the turbine inlet temperature has been continuously escalated, targeting up to 2400 K in state-of-the-art engines [1]. However, this performance enhancement is fundamentally constrained by the temperature limits of structural materials, necessitating advanced thermal protection architectures. As shown in Figure 1, cooled cooling air (CCA) systems represent a critical embodiment, wherein aviation fuel—possessing superior specific heat capacity and density—serves as the ultimate heat sink for compressor bleed air. While this fuel cooling technique significantly improves engine performance, the severe thermal duty imposed on the fuel triggers a complex, multi-stage coking process, as illustrated in Figure 2. The thermal-oxidative coking process is a multi-scale phenomenon, dynamically evolving from gas-phase reactions and physical attachment to surface reactions and layered buildup [2]. While lower temperatures favor autoxidation driven by dissolved oxygen to form amorphous deposits, temperatures exceeding 500 °C trigger thermal cracking that produces dense, graphitic soot [3]. Over time, these deposits mature via sintering, severely impacting the heat transfer properties of the fuel. This tendency for coke deposition constitutes an impediment to the application of fuel cooling, and so improvements in the heat transfer properties and thermal stability of fuel are critical. However, while the macroscopic impact of fuel coking on heat transfer and pressure drop has been widely recognized, the evolution of the coke deposit layer itself—particularly its morphology and chemical composition under realistic fuel cooling conditions—remains insufficiently understood.

Deposit formation in flowing hydrocarbon systems represents a multi-scale, multiphysics phenomenon encompassing: (i) homogeneous gas-phase and liquid-phase oxidation kinetics, (ii) transport of reactive species through boundary layers, and (iii) heterogeneous surface reactions and deposit maturation. Historically, investigations predominantly addressed isolated aspects such as global chemical kinetics [4,5,6] or computational fluid dynamics with simplified chemistry [7,8,9] with limited integration across scales. However, recently, research has rapidly shifted toward multi-physics coupling—integrating thermal, fluid, chemical, and structural dynamics. Recent studies have demonstrated that thermal-oxidative coking is not only temperature-dependent but also highly sensitive to intricate flow structures. For instance, complex geometries like variable cross-sections [10], U-bends [11], and asymmetric ribbed channels [12] have been shown to induce intense local deposition due to strong thermal and flow asymmetries. In recent years, increasing attention has been paid to the mechanisms and suppression of thermal-oxidative coking in aviation fuels, especially under realistic flow and heat transfer conditions. A comprehensive review of jet fuel oxidation stability [13] emphasized that autoxidation dominates deposit formation at moderate temperatures, with kinetics governed by dissolved oxygen level, fuel composition and local thermal gradients. Recent comprehensive studies from the past three years have further elucidated the intricate coking dynamics of RP-3 under supercritical pressures. For instance, incorporating water vapor to enhance secondary flow intensity [14], or utilizing asymmetric truncated ribs in curved cooling channels [12], has been shown to significantly mitigate local heat transfer deterioration and dramatically redistribute oxidative coke deposition. Large-eddy simulations of kerosene in vertical U-tubes [15] further demonstrated that buoyancy-driven secondary flows localize deposits, underscoring the need to integrate real-channel geometry into predictive tools. Meanwhile, systematic morphology studies [16] linked distinct coke textures—granular, filamentous or graphitic—to specific formation pathways and offered simplified kinetic frameworks that predict thickness evolution within ±30%.

Fuel chemistry and modern surrogate modeling have also proven decisive in deciphering coking mechanisms. Over the past three years, researchers have moved beyond simple single-component surrogates to develop advanced multi-component models and virtual fuels that accurately predict aromatic intermediate formation and thermophysical property variations under extreme conditions [17]. Furthermore, reactive molecular dynamics (ReaxFF) simulations have provided unprecedented atomistic insights into the pyrolytic coking pathways of RP-3, revealing the dynamic evolution of carbonaceous networks at the microscopic level [3]. Recent advances in microstructural characterization and reactive molecular dynamics have begun addressing the morphological differences between dense oxidative coke and porous pyrolytic soot [3]. Furthermore, modern surrogate fuel models have rapidly advanced our understanding of both low-temperature oxidation and aromatic intermediate formation [18]. However, despite these strides, existing studies predominantly characterize the static, final deposit states. They largely neglect the dynamic, transient architectural evolution from incipient nucleation to mature film [15], as well as the morphological heterogeneity driven by real-time thermal-hydraulic conditions. At temperatures exceeding ~450 °C—the approximate boundary where pyrolysis rates compete with oxidation rates—mixed morphologies emerge featuring amorphous matrices interspersed with graphitic domains. This bimodal architecture invalidates single-phase effective medium approximations commonly employed in heat transfer models, necessitating multi-scale porosity treatments [19]. A fundamental disconnect persists: macroscopic performance metrics (pressure drop, heat transfer coefficient) and global chemical yields are routinely reported, yet the architectural evolution of the deposit layer—controlling effective transport properties through porosity distribution, tortuosity, and interfacial thermal resistance—remains empirically underconstrained. Consequently, existing predictive models lack morphology-aware closure relations, limiting their utility for design optimization.

To date, the detailed oxidation and deposition processes occurring in a high heat-load flow system have not been completely elucidated. It is necessary to investigate the properties of the deposition layer that forms in the cooling tube to distinguish between different oxidative deposition mechanisms. Because according to previous studies regarding the thermal stability of fuel, oxidation deposition reactions are very complex. The level of dissolved oxygen [20], heating time [21], operational conditions [22,23], fluid dynamics [24], buoyancy [25], heat transfer [26], additives [27] and temperature [28] will all affect the coking characteristics of a system. Specifically, recent systematic evaluations highlight that dissolved oxygen controls the chain initiation pathways [29], while local flow regimes, such as varying Reynolds numbers, dictate whether the deposition is diffusion-limited or temperature-dominated [30]. Unlike previous studies that primarily focus on macroscopic deposition mass, the core novelty of this work lies in explicitly quantifying the coupled evolution of deposit morphology, elemental composition, and residual fuel chemistry under realistic, flow-coupled conditions [1]. By systematically isolating the interacting effects of dissolved oxygen, steep temperature gradients, and exposure time, this study uniquely delineates the transition boundaries between competing autoxidative and sulfur-dominated coking pathways. Ultimately, these specific morphological and compositional findings provide the critical, missing morphology-aware closure relations necessary for next-generation predictive models in actively cooled aero-engine architectures. The present study systematically examines oxidative coking of RP-3 while independently varying dissolved-oxygen concentration, fuel temperature, wall temperature gradient, pressure and exposure time. By coupling post-test SEM, XPS and GC/MS analyses with real-time pressure-drop monitoring, we reveal how deposit morphology, elemental composition and residual fuel composition co-evolve. The resulting data set is intended to benchmark next-generation, morphology-aware coking mechanisms and to provide design guidance for actively cooled injector/heat-exchanger channels operating at ever-increasing turbine inlet temperatures.

## 2. Results and Discussion

### 2.1. The Effect of Dissolved Oxygen Concentration

In the autoxidation regime (dominant for aviation kerosene at 150–450 °C), dissolved oxygen (O_2_) in RP-3 fuel acts as the initiator of free radical chain reactions: O_2_ first reacts with alkyl radicals (R·) generated from hydrocarbon cracking to form alkylperoxyl radicals (ROO·), which further abstract hydrogen from fuel molecules to produce hydroperoxides (ROOH) and new R·—this cyclic chain propagation promotes the formation of alkylperoxides, and their subsequent decomposition (via homolytic cleavage of O–O bonds) generates oxygen-containing intermediates that aggregate into insoluble deposits [9]. Three gradient levels of inlet dissolved oxygen concentration were designed—deoxidized level (<2 ppm), normal level (10 ppm, simulating ambient air-saturated fuel), and high level (32 ppm, simulating extreme oxygen-enriched operating conditions)—to systematically investigate the quantitative and qualitative effects of O_2_ content on the microstructure, particle size distribution, and formation mechanism of oxidative coke deposits.

**Figure 3:**
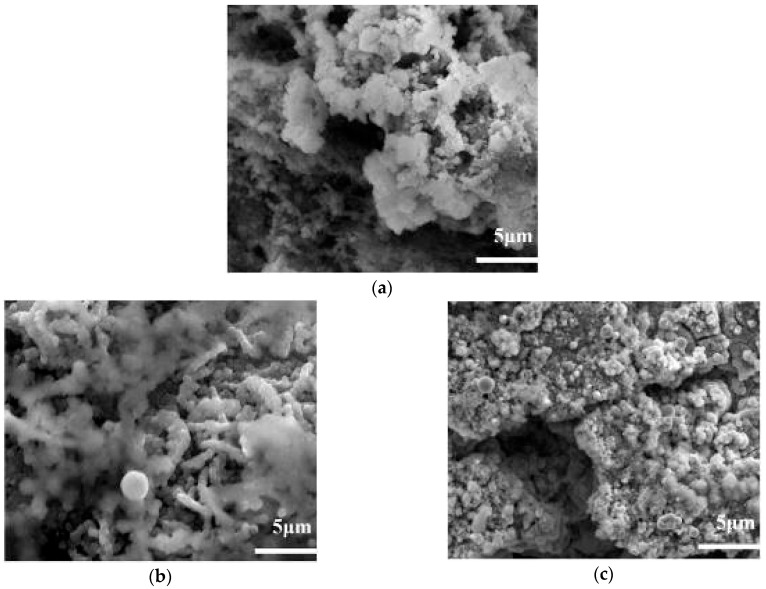
SEM micrographs of inner reactor tube surfaces at various dissolved oxygen concentrations. (**a**) The deposit obtained from fuel with a standard oxygen concentration, (**b**) The deposit obtained with a reduced oxygen concentration, (**c**) The deposit obtained with an elevated oxygen concentration.

Figure 3a presents the SEM micrograph of the deposit at the axial position x = 325 mm (consistent across all trials to eliminate positional interference) under the baseline condition: 410 °C fuel temperature and air-saturated fuel (normal-level O_2_, 10 ppm inlet). High-magnification SEM observation (inset, 60,000×) reveals that the deposit is composed of irregular amorphous and flocculent particles with a loose porous structure, where the particle size ranges from 500 nm to 2 μm and the interparticle voids are attributed to the incomplete aggregation of oxygen-containing intermediates. Similar fluffy, cauliflower-like deposits were also reported by Ervin et al. [31] (for Jet A fuel under 3 MPa and 380 °C) and Tao et al. [32] (for supercritical RP-3), indicating that the flocculent morphology is a universal characteristic of oxidative coke formed via autoxidation-dominated pathways, which is closely related to the stepwise aggregation of soluble macromolecular oxidatively reactive species (SMORS) [19].

Figure 3b,c display the SEM micrographs of deposits at the same x = 325 mm position under deoxidized-level (<2 ppm) and high-level (32 ppm) O_2_ conditions, respectively. Distinct structural differences are observed: the deoxidized sample exhibits a discontinuous, sparse distribution of particles, while the high-O_2_ sample forms a continuous, dense deposit layer—these structural divergences originate from the different reaction pathways triggered by O_2_ availability. Both conditions exhibit morphological heterogeneity indicative of competing formation pathways, but with distinct dominant features reflecting kinetic regime differences. The deposit from deoxidized fuel is significantly looser (porosity estimated to be ~65% via image analysis, compared to ~30% for the normal-level sample) than that from air-saturated fuel, which is attributed to the insufficient dissolved O_2_ limiting the formation of ROOH and SMORS—these oxygen-containing intermediates act as “binding agents” for interparticle aggregation, and their scarcity leads to incomplete particle coalescence and retained voids. Even in the deoxygenated test (initial O_2_ < 2 ppm), deposits are still formed, primarily from the reactions of sulfur-containing species (e.g., benzothiophenes inherently present in RP-3, ~170 ppm [13]) and trace oxygenated impurities (<2% of initial fuel composition). These sulfur-containing species can undergo oxidative coupling reactions in the absence of sufficient O_2_, generating sulfur-oxygen heterocyclic compounds that serve as alternative precursors for deposit formation [33].

A uniform distribution of microscopic needle-like deposits (length: 5–10 μm, diameter: ~200 nm) is clearly observed in Figure 3b, which is uniquely attributed to the coke formation pathway dominated by sulfur-based species. XPS analysis of this deposit confirms a sulfur content of ~3.2%, significantly higher than the normal-level sample (~0.8%), supporting the formation of C-S-H or sulfur-oxygen heterocyclic crystal structures [34].

**Figure 4:**
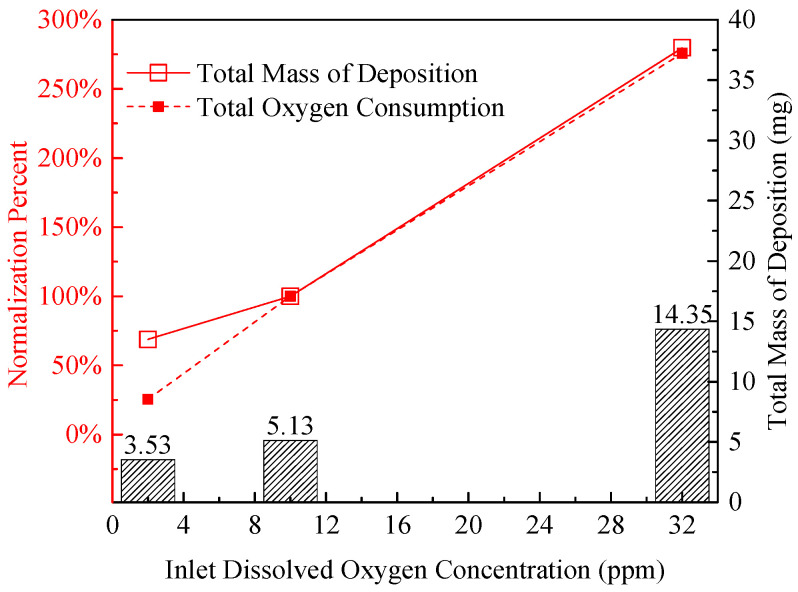
Total deposition mass and oxygen consumption at various inlet dissolved oxygen concentrations.

The coke deposits from high-level O_2_ fuel appear dense (porosity ~15%) and consist of agglomerated large particles (average size ~3 μm), which is attributed to the high O_2_ concentration accelerating the chain propagation of autoxidation: the rapid generation of ROOH and SMORS leads to intensive interparticle collision and fusion, forming compact agglomerates. This observation directly demonstrates that dissolved O_2_ is a rate-limiting factor for the aggregation step in oxidative coking reactions. These distinct microstructural differences (loose needle-like vs. dense flocculent vs. compact agglomerated) directly correspond to the quantitative variations in deposition mass shown in Figure 4. The deoxygenated sample exhibits the lowest mass, the normal-level sample an intermediate mass, and the high-level O_2_ sample the highest—confirming that the microstructure evolution is closely coupled with the extent of deposition. Compared with the amount of coke obtained from the standard oxygen level, the total deposition mass generated by the deoxygenated fuel was decreased by 31% while the elevated oxygen concentration exhibited an 180% increase. This trend of increasing deposition mass with rising O_2_ concentration is further confirmed by the normalized data in Figure 4, where the oxygen consumption (calculated as inlet minus outlet O_2_ concentration) also shows a positive correlation with deposition mass: the high-level O_2_ sample consumes ~18.2 ppm O_2_, the normal-level sample ~6.34 ppm, and the deoxygenated sample <1.5 ppm—indicating that O_2_ consumption is directly proportional to the extent of autoxidation-driven deposition.

**Figure 5:**
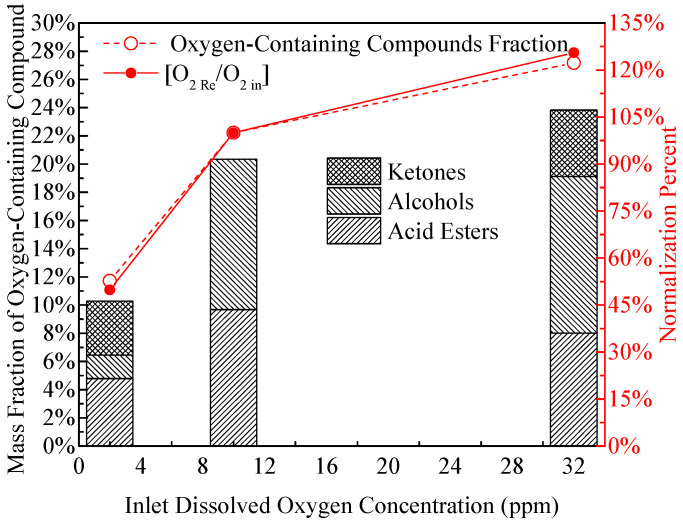
Oxygen-containing compounds in the residual fuel.

Notably, when the inlet O_2_ concentration is reduced to <2 ppm (deoxygenated level), the oxygen consumption drops to ~25% of the normal-level sample (from 6.34 ppm to <1.5 ppm), but the coke deposition mass only decreases to ~68% (0.57 mg/cm^2^ vs. 0.82 mg/cm^2^). This discrepancy suggests that the sulfur-dominated pathway (independent of dissolved O_2_) maintains a non-negligible deposition rate, offsetting ~32% of the reduction in autoxidation-driven deposition. These quantitative data, combined with the SEM observation of sulfur-rich needle-like deposits and GC/MS analysis of sulfur-containing oxygenated compounds (Figure 5), collectively confirm that deposition under low-oxygen conditions is predominantly driven by the reactions of sulfur components (e.g., benzothiophenes) in RP-3 fuel—these species can be oxidized by trace oxygen or metal catalysts (e.g., Fe from the reactor tube) to form reactive intermediates that polymerize into deposits [33,35].

Collectively, the above results demonstrate that varying dissolved oxygen concentrations induce both quantitative (deposition mass, O_2_ consumption) and qualitative (morphology, elemental composition, precursor species) differences in the coke deposit layers. These differences arise from the competition and transition between two dominant deposition pathways: autoxidation-driven (O_2_-dependent) and sulfur-dominated (O_2_-independent) pathways. This result strongly suggests that distinct coking mechanisms operate under different initial dissolved oxygen conditions: (1) under normal/high O_2_ levels, the autoxidation mechanism dominates, with ROOH and SMORS as key precursors; (2) under deoxygenated conditions, the sulfur-catalyzed oxidation mechanism prevails, where sulfur-containing species and trace oxygenated impurities drive deposit formation. Hence, it is essential to further clarify the differences in coke formation mechanisms under varying dissolved oxygen concentrations by integrating the detailed chemical reactions of key species (e.g., R·, ROO·, ROOH, sulfur heterocycles) and their interactions with the reactor wall (e.g., metal catalysis), which can provide a theoretical basis for the development of targeted anti-coking strategies (e.g., deoxygenation vs. desulfurization)

**Figure 6:**
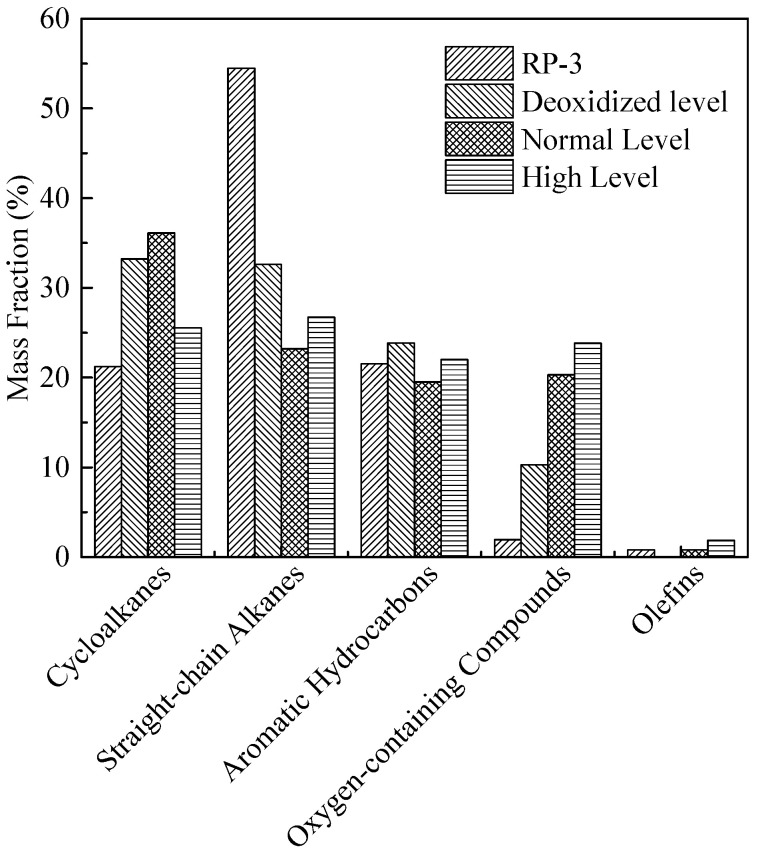
Residual fuel compositions obtained at different dissolved oxygen levels.

Figure 6 presents the GC/MS analysis results of residual fuel composition after coking tests. Quantitative analysis shows that the content of straight-chain alkanes (C_10_–C_16_, the main component of RP-3) decreases by 40%, 57%, and 50% under low, normal, and high oxygen conditions, respectively, compared to the fresh fuel. This trend indicates that straight-chain alkanes are more susceptible to autoxidation than cycloalkanes or aromatics, as their linear molecular structure facilitates hydrogen abstraction by ROO· radicals. Concomitantly, the total content of oxygen-containing compounds in the residual fuel increases significantly by 210%, 480%, and 420% under low, normal, and high oxygen conditions, respectively, relative to fresh RP-3. This substantial increase confirms that oxygen-containing compounds are the direct products of fuel autoxidation, and their concentration is positively correlated with the initial O_2_ level (except for the high-O_2_ sample, where partial thermal decomposition of oxygenates may occur at 410 °C).

**Figure 7:**
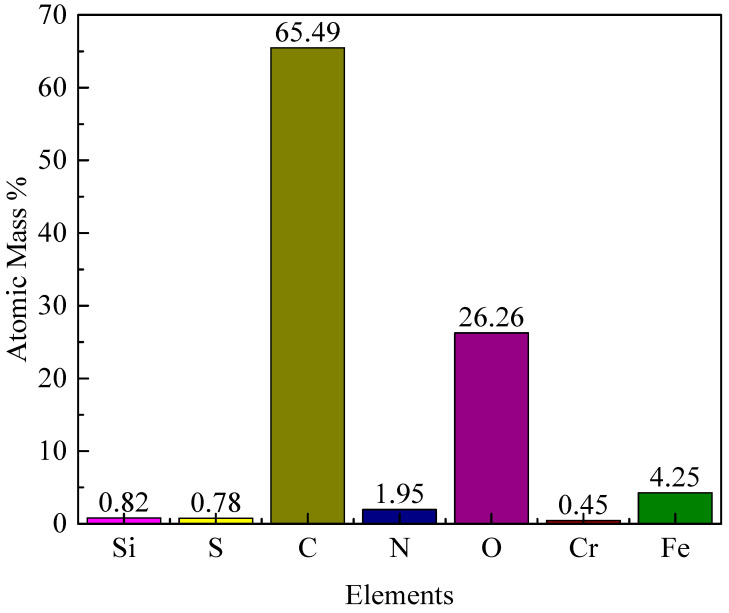
Elemental composition of a coke sample at a normal dissolved oxygen level.

Figure 6 demonstrates that the oxygen-containing compounds consisted of acid esters, alcohols and ketones. Further structural analysis reveals that the oxygen-containing compounds are derived from both straight-chain alkanes (accounting for ~75% of total oxygenates) and cycloalkanes (~25%), with cycloalkanes exclusively converted into cycloalkyl acid esters (e.g., cyclohexyl acetate). This selectivity arises from the higher stability of cycloalkyl radicals, which preferentially react with carboxylic acids (generated from straight-chain alkane oxidation) to form esters rather than undergoing further oxidation to alcohols or ketones. In addition, oxygen-containing compounds containing sulfur were generated at approximately 14.7% and 14% by the low and normal oxygen concentration fuels. In contrast, sulfur compounds accounted for only 5.5% of the output of the high oxygen fuel. These compositional results clearly indicate that when sufficient dissolved O_2_ is available (normal/high levels), the autoxidation of hydrocarbons (straight-chain alkanes in particular) takes precedence as the dominant reaction pathway, generating large amounts of non-sulfur-containing oxygenates (esters, alcohols, ketones) that drive coke formation. Conversely, when dissolved O_2_ is insufficient (deoxygenated level), the oxidation of sulfur-containing heterocycles (e.g., benzothiophenes) becomes the dominant reaction, as these species have higher oxidation potential than alkanes under low-O_2_ conditions, leading to the formation of sulfur-oxygen-carbon heterocyclic compounds that serve as precursors for needle-like coke deposits. This sulfur is likely either initially present in the bulk fuel or comes from the surface of the reaction tube. This effect explains the relationship between the amount of deposition and the fuel sulfur content during the low oxygen trials. The distribution of oxygen-containing compounds in Figure 6 is consistent with the residual dissolved oxygen amounts obtained at varying dissolved oxygen levels. Collectively, the GC/MS analysis of residual fuel, SEM observation of deposit morphology, and XPS elemental analysis (in Figure 7) provide compelling evidence that oxygen-containing compounds (both sulfur-containing and non-sulfur-containing) play a pivotal role as precursors in the oxidative coking mechanism: they undergo further polymerization, cross-linking, and dehydration to form insoluble carbonaceous deposits, with their chemical structure (e.g., linear vs. heterocyclic) determining the final deposit morphology (flocculent vs. needle-like).

Due to the limited amount of deposited material under deoxygenated conditions, reliable XPS characterization was not feasible, while deposits obtained under normal oxygen conditions provided sufficient sample quantity for representative compositional analysis. An XPS analysis of the deposition sample obtained from fuel containing a normal oxygen level gave the results in Figure 7. The normal oxygen condition is considered representative of practical operating scenarios, whereas elevated oxygen levels are primarily used to illustrate the trend of oxygen-enhanced deposition behavior. These data show relatively high C and O levels of 65.49 and 26.26%, respectively. In a previous study of deposition, Huang et al. [36] assumed a deposit density of 1 g/cm^3^ (less than that of black carbon but similar to soot) to predict the coke thickness [37]. However, it is obvious that the coke deposition layers formed in the current study were composed of other elements in addition to carbon. Elements obtained from the reaction tube, such as Fe, Ni and Co, are widely believed to promote coke deposition [35]. For example, the XPS data show an Fe concentration of 4.25 at.% in the normal-level deposit, which is significantly higher than the surface Fe concentration of the fresh reactor tube (~0.3 at.%), indicating that Fe is selectively adsorbed and enriched on the deposit surface during coking. This enrichment confirms the metallic catalysis effect: Fe active sites facilitate the decomposition of ROOH into alkoxy radicals (RO·) and hydroxyl radicals (·OH), which further accelerate the polymerization of fuel molecules into coke.

### 2.2. Effect of Varying Temperatures

**Figure 8:**
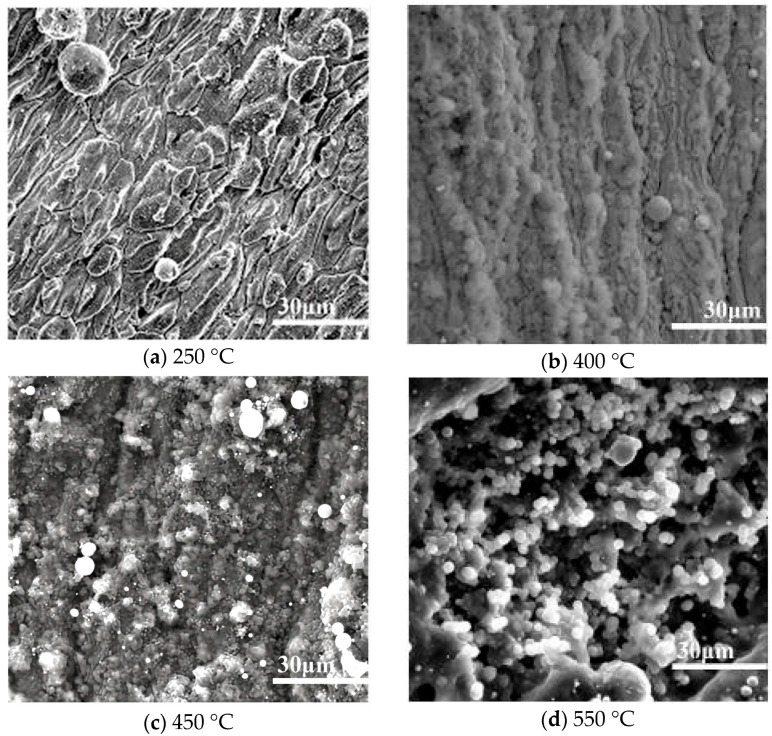
SEM micrographs of depositions obtained at different fuel temperatures.

Temperature is universally recognized as one of the most critical kinetic factors determining the rate of oxidative coking, as it regulates both the thermodynamics (reaction equilibrium) and kinetics (rate constants) of autoxidation and pyrolysis reactions, and further modulates the microstructure and chemical composition of coke deposits. Figure 8 presents SEM micrographs of coke deposits collected from different axial positions along the reactor, where each position corresponds to a specific fuel temperature (250 °C, 400 °C, 450 °C, 550 °C) due to the axial temperature gradient of the reactor. This sampling strategy allows for systematic investigation of temperature effects while maintaining other experimental parameters (O_2_ concentration: 10 ppm; pressure: 3 MPa; duration: 1.75 h) constant. Comparing these images with the pristine surface (as shown in the Method section) indicates that no obvious changes are observed in the sample exposed to the low temperature of 250 °C (Figure 8a). However, the 60,000× micrograph does demonstrate the presence of some fluffy particles, corresponding to the low deposition rate at low temperatures.

**Figure 9:**
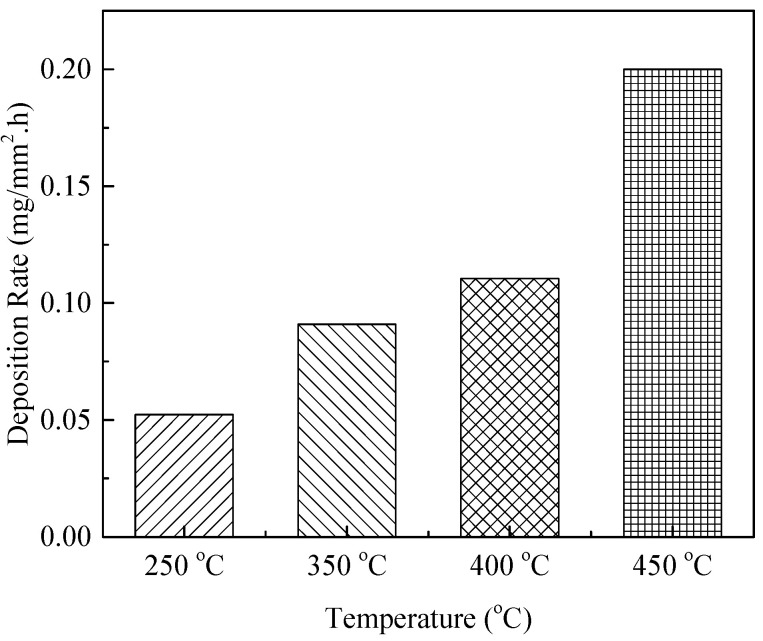
Deposition rates at various temperatures.

The deposit obtained at 400 °C is shown in Figure 8b; this deposit is denser than the typical SEM micrograph in Figure 3a. In addition, the deposition rate underwent an obvious increase with increasing temperature, as shown in Figure 9. The deposition process is dominated by autoxidation reactions below 450 °C, while above this point both pyrolysis and oxidation reactions take place simultaneously, as seen in Figure 8c, and a typical oxidation deposition tends to contain some amount of spherical particles. Pyrolysis reactions are also known to involve the breakdown and recombination of hydrocarbon chains, resulting in deposits [38,39], and the pyrolysis reaction rates are generally faster than the oxidation deposition rates. Figure 8d presents a SEM micrograph of pyrolysis deposition generated at a temperature of 550 °C. This deposit has an entirely different structure from the oxidation deposition and is composed of spherical submicron particles with an average diameter of 1–2 μm.

**Figure 10:**
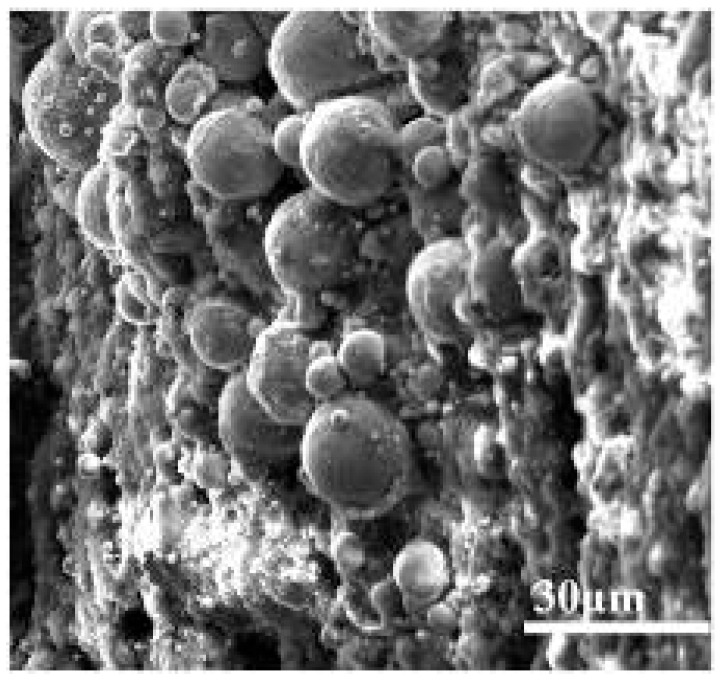
SEM micrograph of the inner surface of the reactor tube in a high temperature gradient section.

In addition to the absolute temperature, the temperature gradient (*dT*/*dx*) along the reactor tube also significantly affects the deposition rate and microstructure, as the rate of temperature change modulates the local concentration gradient of fuel oxidation products and the mass transfer of precursors to the tube wall. The axial temperature gradient of the reactor varies from ~0.5 °C/mm (low gradient region) to ~1.5 °C/mm (high gradient region) due to the reactor heating design. Our previous studies demonstrated that significant deposits were formed in areas experiencing high temperature gradients during the temperature range associated with thermal oxidation deposition [40]. The micrograph in Figure 10 shows that deposits in the high temperature gradient (*dT*/*dx* = 1.5 °C/mm) section were composed of spherical submicron particles with an average diameter of 10–15 µm. These particles were therefore larger than those resulting from pyrolysis deposition. These results show that very different deposits can be generated in the high temperature gradient region and thus demonstrate the important role of temperature gradients in coke formation. This temperature gradient effect should be explicitly incorporated into deposition mechanisms by accounting for the coupling between temperature gradient-induced mass transfer (secondary flow) and precursor aggregation kinetics, which will improve the accuracy of coking prediction models for practical aero-engine cooling channels where axial and radial temperature gradients are inherent. The diverse microstructures formed during deposition can produce complex porosity and thermal contact resistance distributions, further affecting the heat transfer characteristics of the deposit. Collectively, these observations confirm that temperature affects the oxidative coking process through multiple pathways: (1) regulating the kinetics of autoxidation and pyrolysis reactions; (2) modulating the microstructure and porosity of deposits; (3) inducing temperature gradients that enhance mass transfer and precursor aggregation. Consequently, temperature is a multi-dimensional critical factor that influences both the deposition mechanism and the subsequent heat transfer performance of the fuel cooling system.

### 2.3. The Effects of Varying the Pressure

**Figure 11:**
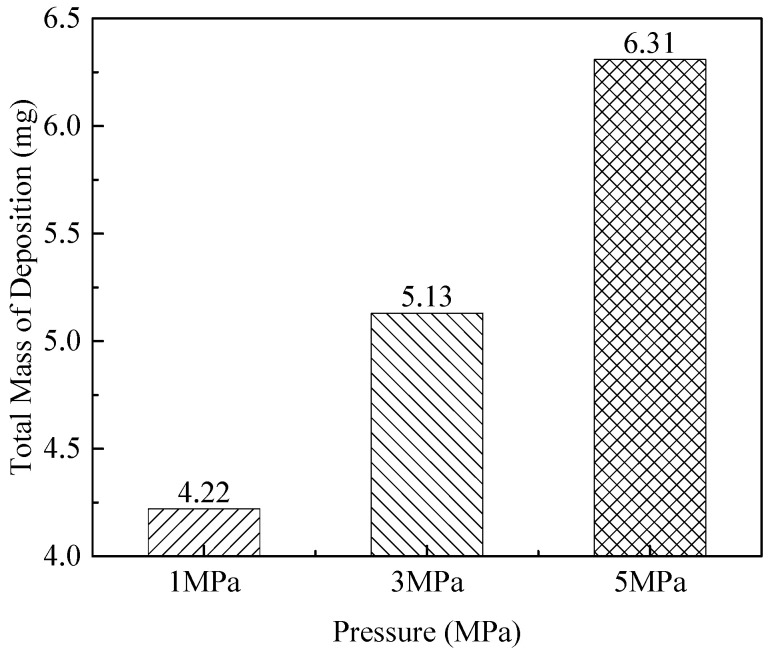
Total deposition masses obtained at different pressures.

The operating pressure of a typical aircraft fuel system ranges from 3.45 to 6.89 MPa. Therefore, it is important to investigate oxidative deposition properties at various pressures. In this study, the effects of system pressure on deposition were examined using air-saturated fuel at a constant wall temperature. The total deposition masses resulting from these trials at three different pressures are provided in Figure 11. It can be seen that the total amount of deposition increases somewhat with an increase in pressure. In comparison with the typical 3 MPa pressure, the deposit mass was reduced by 17% at a lower pressure of 1 MPa, and increased by 23% at 5 MPa. Therefore, relative to the effects of temperature and oxygen, varying the pressure has a minimal effect on the deposition rate.

**Figure 12:**
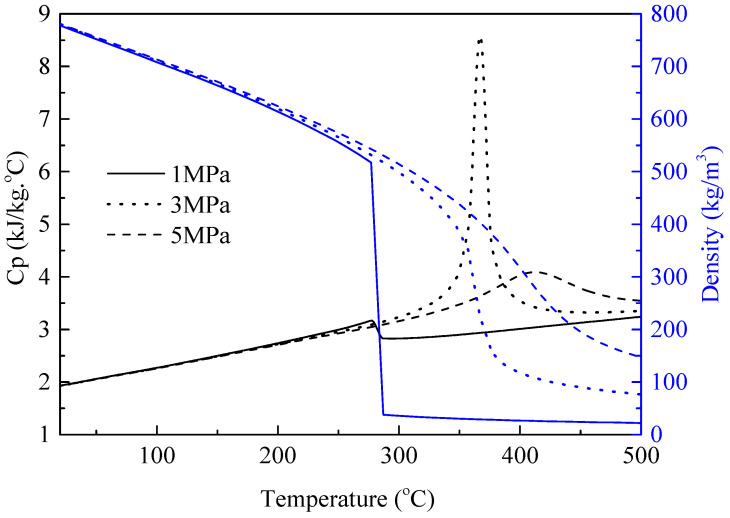
Variations in specific heat and density with temperature at different pressures.

**Figure 13:**
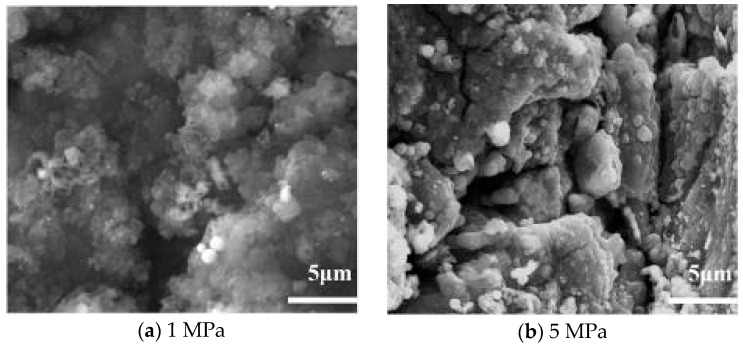
SEM micrographs of deposits obtained at different pressures.

Figure 12 summarizes the specific heat and density values calculated using a three-component surrogate model [41]. It is apparent that these two parameters both change significantly with pressure, especially near the critical temperature. Therefore, the primary effect of pressure on oxidative deposition is indirect, mediated by changes in fuel thermophysical properties (*c*_*p*_, *ρ*) and flow conditions: higher pressure increases fuel density and viscosity, leading to a higher Reynolds number (*Re*) and enhanced convective heat transfer, which in turn accelerates the transport of oxygen and fuel molecules to the tube wall—promoting surface reactions and deposition. The micrograph of the deposit obtained at 1 MPa in Figure 13a is similar to that obtained at 3 MPa in Figure 3a, although slightly less dense.

In contrast, Figure 13b (5 MPa) shows a dense and compact deposit structure with a low porosity (~22%) and large agglomerated particles (~1.2 μm). This compactness arises from the higher fuel density and viscosity at 5 MPa, which increase the collision frequency and adhesion efficiency of oxygen-containing precursors, promoting particle fusion and densification—consistent with the indirect effect of pressure on deposition structure.

**Figure 14:**
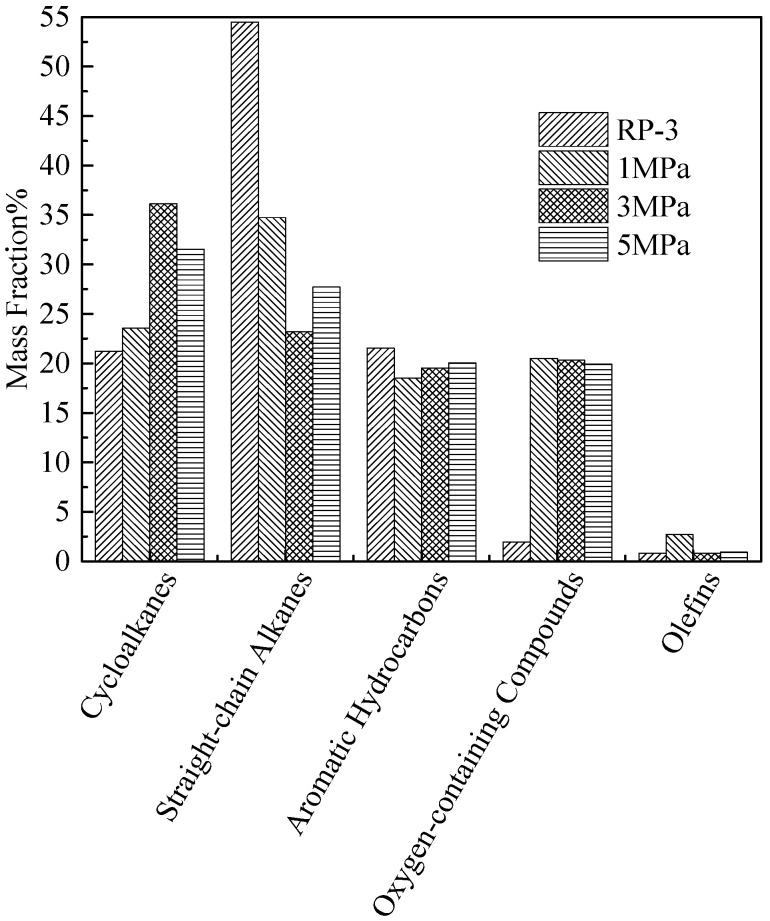
The fuel composition at varying pressures as determined by GC-MS.

The results of the composition analysis of the liquid residual fuel at different pressures are summarized in Figure 14.

Consistent with the oxygen concentration variation results, all pressure conditions show a decrease in straight-chain alkanes (48–57% reduction relative to fresh RP-3) and an increase in cycloalkanes (~15–20% increase) and oxygen-containing compounds (~380–450% increase). This consistency indicates that pressure does not alter the dominant autoxidation pathway of RP-3, as straight-chain alkanes remain the primary species undergoing oxidation.

Furthermore, the type of oxygen-containing compounds (acid esters, alcohols, ketones) remains unchanged across all pressure conditions, confirming that pressure does not affect the reaction pathways of autoxidation (e.g., esterification, alcohol formation, ketonization). This observation further supports the conclusion that pressure has a limited effect on the chemical mechanism of oxidative deposition, acting primarily through physical rather than chemical pathways. The oxidative deposition process is governed by two key steps: (1) bulk fuel autoxidation (formation of oxygen-containing precursors), which is dominated by chemical kinetics (O_2_ concentration, temperature); (2) wall deposition (adsorption and aggregation of precursors), which is regulated by physical factors (mass transfer, precursor collision frequency). Changes in pressure primarily affect the second step (wall deposition) by modifying fuel flow and mass transfer, while having no significant impact on the first step (bulk autoxidation)—this explains the limited effect of pressure on overall deposition.

### 2.4. Effect of Heating Duration

**Figure 15:**
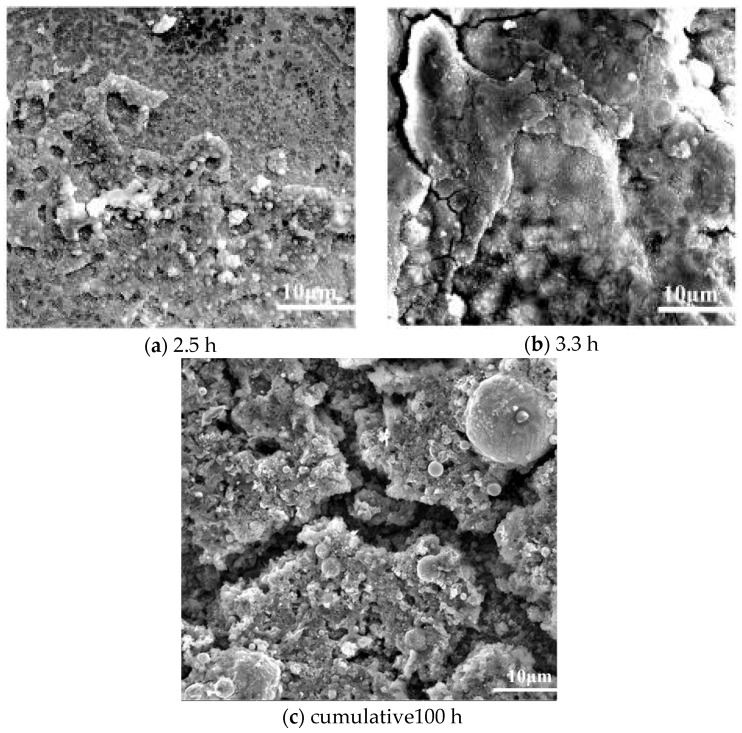
SEM micrographs of samples following prolonged test durations.

The effects of heating duration on the deposition microstructure are characterized in Figure 15, covering both short-term continuous tests and long-term cumulative operating conditions. For short-term trials, extending the test duration from 1.75 h to 2.5 h maintains the porous nature of the deposits (consistent with the morphological features in Figure 3a), where the porous structure enlarges the contact area between the fuel and the reactor wall, providing abundant active sites for the adsorption and aggregation of oxygenated colloidal precursors and thus stimulating further coke formation. As the duration is prolonged to 3.3 h, the porous deposits gradually undergo sintering and densification, transforming into hardened compact structures that fully cover the inner surface of the 316 stainless-steel (SS316) tube. This hardened layer acts as a physical barrier, separating the fuel from the metal wall and inhibiting the metal-catalyzed coking reaction driven by Fe/Ni active sites on the SS316 surface. Consequently, the deposition rate becomes moderate due to the combined insulating effect (reducing interface heat transfer efficiency) and catalytic site blocking effect of the accumulated coke layer—consistent with the findings of Marteney’s research on JP-5 fuel [42]. With the continuous accumulation of coke, the effective flow cross-sectional area of the reactor tube decreases, leading to an increase in fuel flow velocity and a corresponding pressure drop, which further modifies the mass transfer behavior at the fuel–wall interface. Therefore, to accurately predict the dynamic evolution of oxidative coking over extended durations, deposition kinetic models must incorporate not only chemical kinetics (autoxidation, metal catalysis) but also time-dependent physical factors: (1) mass transfer changes due to flow velocity increase; (2) deposit thickness-dependent catalyst site blocking; (3) thermal insulation-induced wall temperature reduction. This integrated model will better reflect the practical coking behavior in aero-engine cooling channels, which operate for extended periods.

To simulate the actual service conditions of aero-engine fuel cooling systems (which involve repeated startup-shutdown cycles and fuel flushing), a long-term cumulative test of 100 h was also conducted, involving periodic cooling, flushing, and reheating processes. The microstructure of the coke deposits after 100 h of cumulative operation exhibits distinct characteristics compared to short-term continuous tests: in addition to large, dense structural aggregates that are tightly bonded to the tube wall, the overall deposit morphology shows obvious signs of mechanical damage and selective retention. This is attributed to the synergistic effects of thermal cycling and fluid scouring during the long-term cumulative process: repeated heating and cooling induce thermal expansion and contraction mismatches between the coke layer and the SS316 substrate, generating microcracks in the deposits; meanwhile, the continuous fuel flow exerts shear stress on the coke surface, further propagating these cracks and causing loose, poorly bonded structural fragments to detach and be carried downstream. As a result, only the coke structures with high mechanical strength and strong interface bonding to the tube wall are retained, while the fragile or loosely attached deposits are gradually removed. This dynamic “formation-damage-retention” process leads to a non-linear relationship between coke accumulation and time—unlike the relatively regular evolutionary trend observed in short-term continuous tests, the long-term cumulative deposition behavior is significantly regulated by cyclic operating conditions (e.g., thermal shock, fluid scouring) and the structural stability of the coke itself.

Furthermore, the repeated heating-cooling cycles may alter the chemical environment at the fuel–coke interface: the thermal shock-induced cracks expose fresh coke surfaces and underlying metal sites during reheating, providing new active centers for the adsorption of oxygenated precursors in the fuel; at the same time, the flushing process removes some soluble intermediates and loose coke particles, reducing the competition for active sites and potentially affecting the subsequent deposition kinetics. This complex dynamic evolution of the coke layer during long-term cumulative operation highlights that the coking process in practical engineering systems is not a simple linear extension of short-term reactions, but rather a comprehensive result of the coupling of chemical reactions (oxidation, polymerization), physical processes (sintering, thermal shock, fluid scouring), and interface interactions (coke-wall bonding, precursor adsorption). Therefore, when designing fuel cooling channels and formulating anti-coking strategies for aero-engines, it is necessary to fully consider the long-term cyclic operating conditions and the non-linear growth characteristics of coke deposits, and incorporate factors such as structural stability of deposits and interface bonding strength into the predictive model.

## 3. Methods

**Figure 16:**
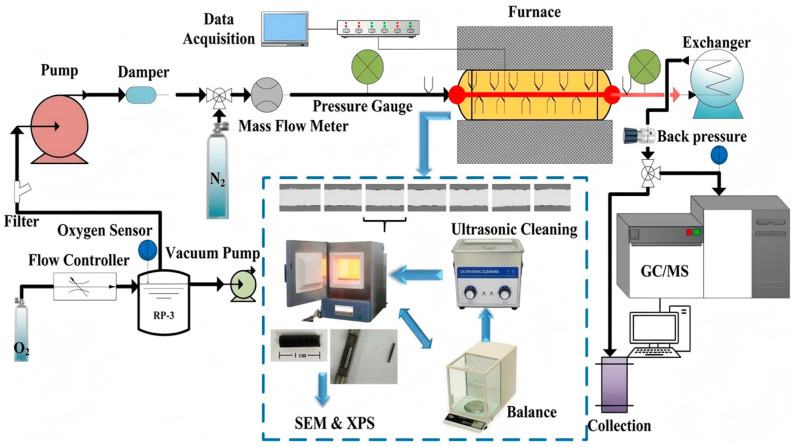
The experimental setup.

A standard Chinese aviation kerosene (RP-3) was employed as the fuel in this study [41]. RP-3 is primarily composed of straight-chain alkanes (54.4 wt%), cycloalkanes (21.3 wt%) and aromatic hydrocarbons (21.5 wt%). An indirectly electrically heated reactor was used to simulate the heat exchanger in an aero-engine cooling system. with the test section specifically fabricated from 316 stainless-steel tubing. This material was selected for its superior corrosion resistance compared to conventional stainless steels (e.g., 304), which is particularly critical for minimizing systematic errors during subsequent coke mass measurement via acid washing. The enhanced corrosion resistance of 316 stainless steel prevents excessive material loss or surface degradation caused by the acid cleaning process, ensuring the accuracy of coke mass calculations with relatively low relative errors. During each trial, the fuel was heated with the flow process and thermal oxidation deposition reactions occurred simultaneously. Details of the experimental setup and measurement systems are provided in Figure 16. The experimental setup comprised four main subsystems: fuel supply, heating and reaction, data acquisition and control, and sampling/online analysis. Prior to testing, the fuel was deoxygenated using a vacuum pump and magnetic stirring. The dissolved oxygen level was continuously monitored using an in situ oxygen sensor connected to a microprocessor transmitter, and subsequently adjusted to the desired concentration via a mass flow controller. The conditioned fuel was filtered and delivered to the reactor at a constant mass flow rate of 1 g/s, with flow and pressure stabilized by a metering pump, Coriolis flow meter, and pressure damper. The test section consisted of a horizontal 316 stainless-steel tube, externally heated to impose a controlled heat flux. Downstream of the reactor, the fuel was cooled through a counter-flow heat exchanger, and system pressure was maintained at 3 MPa using a back-pressure regulator to ensure supercritical conditions. Due to the elevated temperature and pressure at the reactor outlet, oxygen concentration measurements were conducted after cooling via a dedicated sampling and online analysis unit. System sealing integrity was verified by comparing inlet and outlet oxygen concentrations. Pressure drop across the reactor was measured using a differential pressure transducer, while the outer wall temperature was monitored by an array of thermocouples distributed along the tube. Inlet and outlet fuel temperatures were measured at the tube centerline. Each experiment was conducted for 105 minutes to ensure sufficient deposition for subsequent analysis. The oxygen concentration, fuel mass flow rate, pressure, and fuel and wall temperatures were all monitored by a data acquisition system. More details regarding the experimental setup and measurements can be found in our previous publications [20,23,40].

Following each coking experiment, the straight stainless-steel tube (inner diameter of 2 mm and length of 450 mm, surface roughness Ra < 0.4 μm) was cut into equal parts, followed by vacuum drying in an oven, ultrasonic cleaning and weighing on a high precision balance to obtain data regarding mass deposition along the reactor [40]. Deposit morphology was characterized by field-emission SEM (FEI Quanta 650 FEG, FEI Company, Hillsboro, OR, USA, 5 kV accelerating voltage, 10 mm working distance) at a fixed axial position (x = 325 mm, corresponding to T_fuel_ ≈ 380–400 °C under standard conditions), ensuring comparability across trials. This location was selected based on the following criteria: (i) it corresponds to a local bulk fuel temperature of approximately 380–400 °C under baseline conditions, which lies within the autoxidation-dominated regime; (ii) it consistently represents the region with the highest deposition activity across all experimental cases; and (iii) the use of a fixed axial position minimizes positional variability and ensures reliable comparisons between different operating conditions. In the majority of previous experimental studies, morphological analysis has been used to observe the detailed microstructure of the deposition layer as well as to determine coke particle sizes. Although microphotography is sufficient to identify differences in structures, it cannot provide quantitative results. Therefore, a 1 cm section of the tube was cross-sectioned by wire-electrode cutting to allow for surface elemental analysis by X-ray photoelectron spectroscopy (ESCALAB 250Xi, Thermo Fisher Scientific, East Grinstead, UK, monochromatic Al Kα source, 20 eV pass energy). The relative standard deviation (RSD) for the elemental content was found to be within 8%, which is consistent with the instrumental precision of the spectrometer. Data regarding the residual fuel composition were also necessary to investigate bulk fuel reactions, and these compositions were obtained by gas chromatography/mass spectrometry (GC/MS, 7890B–5975A, Agilent Technologies, Santa Clara, CA, USA). The Agilent 7890B–5975A system was operated in Split mode (10:1) with a constant flow of Helium (1.0 mL/min). Repeated injections of a standard check solution showed a relative standard deviation (RSD) of less than 5% for peak areas, demonstrating excellent instrumental stability. The residual fuel samples were collected after each experiment and the organic phase was then filtered through a 0.22 μm PTFE membrane before injection to remove suspended solid deposits. Quantification was performed using the external standard method. A five-point calibration curve was constructed using certified reference standards for major fuel components. The correlation coefficients (*R*^2^) for all calibration curves were greater than 0.995, ensuring the accuracy and linearity of the quantitative results. The GC/MS analysis in this study focuses on the comparative variation of major species groups under different operating conditions.

The various oxygen concentrations applied in this work are summarized in Table 1. In each case, the heat flux, time, pressure and mass flow rate were kept constant. The effects of temperature were examined by assessing different sections along the reactor, corresponding to varying fuel temperatures, while effects of pressure on deposition were determined by varying the pressure at the same wall temperatures. The effects of the test duration were investigated by extending the experiment time while maintaining a constant fuel temperature at the outlet of the reactor. The temperature gradient along the reactor was determined using the S-tube test [43].

**Figure 17:**
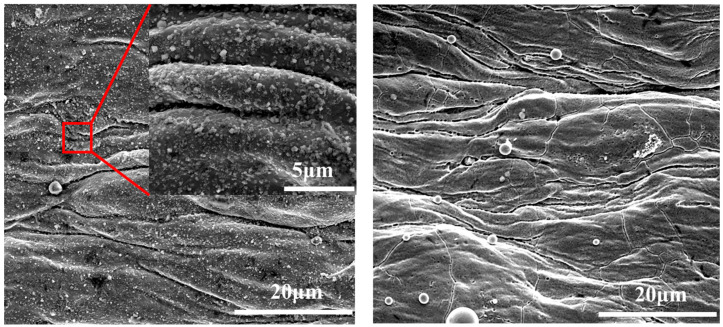
SEM micrograph of an inner reactor tube surface prior to coking experiments and post-decoking condition.

To facilitate data comparisons, the inner surface of a reactor tube before a coking trial was analyzed by SEM and a typical image is shown in Figure 17. The untouched surface exhibits random ravines and embossments. A complicated structure such as this, having an enlarged surface area, will tend to promote the deposition of insoluble substances.

## 4. Conclusions

This study systematically investigated the oxidative coking behavior of RP-3 aviation kerosene under representative fuel cooling conditions. The main findings are summarized as follows:

1. Dissolved oxygen governs both the extent and mechanism of oxidative coking in the autoxidation regime. Increasing O_2_ concentration significantly enhances deposition mass, with increases reaching up to 180%, and promotes the formation of dense flocculent deposits. In contrast, deoxygenation reduces deposition by approximately 31% but does not fully suppress it. Two distinct pathways are identified: under oxygen-rich conditions, autoxidation dominates and generates oxygen-containing intermediates that form flocculent deposits; under oxygen-deficient conditions, sulfur-containing species dominate, leading to needle-like structures.
2. Temperature controls both deposition rate and morphology, with a clear regime transition. Below ~450 °C, oxidative deposition dominates, while above this temperature pyrolysis contributes significantly, producing spherical and graphitic structures. Steep temperature gradients further induce localized deposition and distinct microstructures.
3. Pressure has a secondary and indirect influence on coking behavior. Increasing pressure leads to moderate changes in deposition, with a reduction of about 17% at 1 MPa and an increase of about 23% at 5 MPa compared with the baseline condition of 3 MPa. This effect is primarily associated with variations in fuel thermophysical properties and mass transfer, while the dominant chemical pathways remain unchanged.
4. Deposition exhibits strong time-dependent and non-linear evolution. Initial porous deposits promote further growth, whereas prolonged operation leads to densification, catalyst-site blocking, and reduced growth rate. Under cyclic conditions, deposition is governed by the combined effects of growth, thermal damage, and fluid-induced removal.
5. Deposit morphology is a key factor linking operating conditions to coking behavior and heat transfer performance. Morphology determines porosity, interfacial thermal resistance, and catalytic-site accessibility. The strong dependence of deposit structure on oxygen availability, temperature gradients, and operating history indicates that morphology-aware descriptions are essential for accurate prediction and design of fuel-cooled aero-engine systems.

## Figures and Tables

**Table 1:** Oxygen concentrations applied during various trials.

Cases	Inlet Oxygen Concentration/ppm	Outlet Oxygen Concentration/ppm	Heat Flux /kW/m^2^	Heating Time /hour	Mass Flow Rate /g/s
Deoxidized-level	<2	<0.5	1	1.75	1
Normal-level	10	3.66			
High-level	32	13.8			

## Data Availability

The data that support the findings of this study are available from the corresponding author [Xinyan Pei] upon reasonable request.
